# Sodium and Water Homeostasis in Children: Pathogenesis, Diagnosis, and Treatment

**DOI:** 10.3390/jcm15020852

**Published:** 2026-01-20

**Authors:** Monika Dąbek, Michał Szyszka, Piotr Skrzypczyk

**Affiliations:** 1 Student Scientific Group, Department of Pediatrics and Nephrology, Medical University of Warsaw, 02-091 Warsaw, Poland; 2 Department of Pediatrics and Nephrology, Doctoral School, Medical University of Warsaw, 02-091 Warsaw, Poland; 3 Department of Pediatrics and Nephrology, Medical University of Warsaw, 02-091 Warsaw, Poland; pskrzypczyk@wum.edu.pl

**Keywords:** sodium, syndrome of inappropriate antidiuresis, cerebral salt wasting syndrome, central diabetes insipidus, nephrogenic diabetes insipidus, treatment, diagnosis

## Abstract

Maintaining homeostasis in the body through water and sodium management is essential, and the central nervous system and kidneys play a key role in this process. However, knowledge of the diagnosis and treatment of these conditions in pediatric patients is still unsystematized. There are no up-to-date guidelines on managing children with sodium imbalance. Since sodium shifts are inextricably linked to water changes in the body, they should always be pondered together. Each of the sodium disorders should be considered in the context of changes in the vascular volume, whether it is hypo-, eu-, or hypervolemic. This review describes the most common sodium-water disorders encountered in pediatric clinics. It emphasizes conditions affecting the brain-kidney axis (syndrome of inappropriate antidiuresis, cerebral salt wasting syndrome, and central and nephrogenic diabetes insipidus). The article proposes diagnostic and therapeutic management based on scientific society publications, case series, and the authors’ clinical experience, and summarizes the available knowledge as of 2025 to improve the care of patients with hyponatremia or hypernatremia. A proper understanding of the physiology of sodium homeostasis is crucial for implementing appropriate treatment and reducing the risk of severe complications in young patients in the future.

## 1. Introduction

Water-sodium balance is essential for maintaining the healthy functioning of the body. It is preserved by proper absorption in the intestines and excretion through urine, feces, and the skin [1]. The regulation of sodium intake and excretion occurs at three levels: behavioral, cerebral, and renal, and it is triggered by even a slight fluctuation in the amount of sodium in the body [2]. The HPA (hypothalamic–pituitary–adrenal) axis and the kidneys cooperate to maintain appropriate plasma osmolarity [3]. The kidneys play a significant role in maintaining the proper amount of sodium ions in the body, as this is the primary path through which they are excreted. They primarily regulate sodium homeostasis through hormonal control of tubular sodium reabsorption. Activation of the renin–angiotensin–aldosterone system (RAAS) enhances sodium retention via angiotensin II-mediated proximal tubular reabsorption and aldosterone-dependent sodium transport in the distal nephron [4]. Conversely, natriuretic peptides promote sodium excretion by inhibiting RAAS activity and reducing tubular sodium reabsorption [5]. These opposing hormonal influences enable the precise regulation of extracellular volume and blood pressure. The central regulation of sodium and water balance is mediated by hypothalamic osmoreceptors and baroreceptor inputs, which control the secretion of antidiuretic hormone (ADH) and thirst. ADH increases renal water reabsorption [6]. Its integration with the renin–angiotensin–aldosterone system (RAAS) and sympathetic pathways links central sensing to renal sodium and water handling.

This review focuses on the pathophysiology of the key regulatory axes responsible for sodium and water homeostasis in the human body, particularly the brain-kidney axis, with a particular focus on pediatric patients. We discuss the neurohormonal and renal mechanisms involved in regulating sodium balance, including central sensing pathways and renal effector systems. We also summarize the most common clinical disorders associated with dysnatremia, specifically hyponatremia and hypernatremia, and highlight their underlying mechanisms. Lastly, we present current diagnostic approaches and therapeutic strategies for the evaluation and management of the most common and clinically challenging disorders of sodium balance. Understanding the complexity of water-sodium regulations and the complementary roles of both the kidneys and the brain may be the key to proper fluid management in many widespread children’s diseases.

### 1.1. Sodium and Water Crosstalk in the Human Body

Sodium is one of the basic elements that we obtain from our diet. Its content is maintained at approximately 60 mmol/kg body weight [7]. Part of the sodium ions in the body belong to the exchangeable fraction (70% of sodium ions)—those are ions that move by diffusion between the plasma, the intracellular space, the extracellular space, and into the exchangeable part of the bone and cartilage tissue. The remaining 30% is a non-exchangeable fraction associated with both hard and soft tissues [1]. International guidelines recommend age-adjusted limits on sodium intake for children, reflecting their lower energy needs compared to adults [8]. These limits lower the maximum recommended intake value of 2 g/day. The recommended sodium intake for children increases with age: from 110–370 mg/day for infants to 800 mg/day for children ages 1–3 to 1000 mg/day for children ages 4–8 to 1200 mg/day for boys and girls ages 9–13 and to 1500 mg/day for adolescents ages 14–18 [9]. Nearly 90% of sodium consumed comes from salt (sodium chloride) [10]. Most of this element (98–99.9%) is excreted in the urine, and the remainder is reabsorbed by the kidneys [1]. The exact amount that has been filtered by the kidneys and excreted in the urine is provided by the fractional sodium excretion index [11], calculated according to the formula [12]:

$${\mathrm{FE}}_{\mathrm{Na}}\;=\;100\;\times \;\frac{\left[Na\right]urinary\times serum\;creatinine}{\left[Na\right]serum\times urinary\;creatinie}[\%]$$

**Figure 1:**
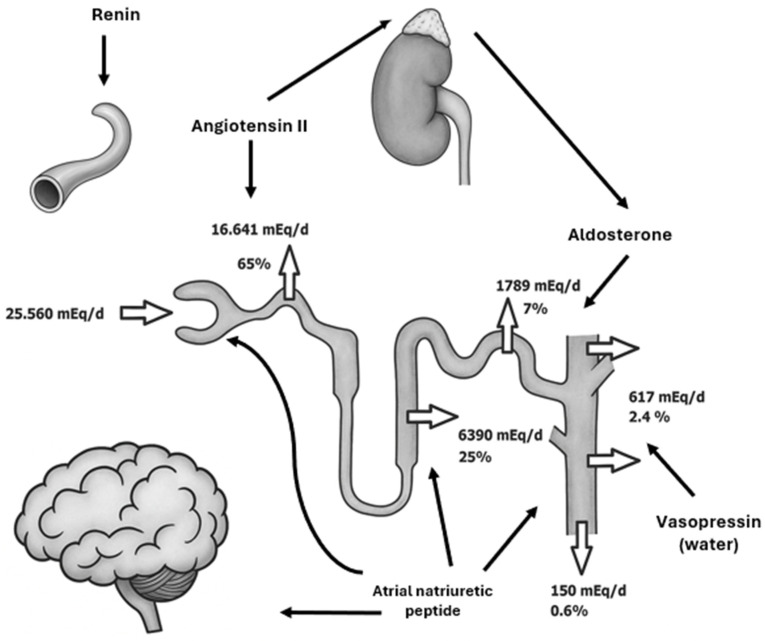
Renal tubular sodium reabsorption in healthy individuals, as well as the main hormones and enzymes that influence it and their sites of action (or receptors). Renin converts angiotensinogen into angiotensin I in the plasma of blood vessels. Angiotensin II promotes the release of aldosterone from the adrenal cortex and acts directly on proximal tubules, promoting Na^+^ reabsorption and H^+^ excretion. Aldosterone acts on nuclear mineralocorticoid receptors (MRs) in the distal tubule and collecting duct, thereby upregulating and activating basolateral Na^+^/K^+^ pumps. Aldosterone also upregulates epithelial sodium channels (ENaCs) in the collecting duct, thereby increasing sodium absorption. ANP affects sodium channels in collecting ducts and directly dilates the afferent arteriole of the nephron. It also decreases sodium reabsorption in the thick ascending limb (via interaction with NKCC2) and the cortical collecting duct of the nephron. ANP also inhibits vasopressin secretion in the hypothalamus. Vasopressin increases passive water transport in renal collecting tubules and collecting ducts, thus indirectly lowering sodium concentration in the plasma. White arrows indicate sites of sodium reabsorption and excretion, whereas black arrows denote the sites of action.

This indicator is based on the comparison of the amount of sodium excreted in the urine to the total amount of the element that is filtered by the kidneys [11]. Neurons in the subfornical organ (which plays a crucial role in sodium sensing) and organum vasculosum of the lamina terminalis are responsible for the appetite for salty foods [13]. Similarly, angiotensin II [13] and aldosterone stimulate the desire to eat salty dishes [14]. Sodium ions are mostly (66% of all) reabsorbed in the proximal tubule of the nephron in active transport by ATPase and partially by passive diffusion,;25% of sodium ions are absorbed in the loop of Henle, and about 6% in the distal tubule and collecting duct altogether [15], as shown in Figure 1. The kidneys are sympathetically innervated by adrenergic neurons originating from the celiac plexus, thoracic splanchnic nerves, and superior mesenteric ganglion, which terminate in the walls of the vessels of the cortex and medulla, the walls of the proximal tubules, the juxtaglomerular apparatus, both arms of the loop of Henle, and the distal tubules and collecting ducts [16]. Neuromodulators with a stimulating effect include noradrenaline, angiotensin II, and bradykinin [17]. Dopamine and natriuretic peptides have an inhibitory effect [18]. Noradrenaline reduces diuresis, increases sodium ion retention by activating Na-K ATPase, and stimulates renin secretion and the activation of the RAA system [19]. Activation of the sympathetic nervous system leads to the inhibition of diuresis, resulting in lower sodium excretion by the kidneys. It is worth emphasizing that in the case of renal denervation, sodium excretion increases [20]. These processes are controlled by specialized regions within the central nervous system that coordinate the integration of autonomic inputs and the modulation of sympathetic outflow. These neural structures assimilate signals originating from peripheral receptors as well as higher-order central pathways, subsequently generating coordinated patterns of autonomic activity. Through this hierarchical organization, the central nervous system ensures precise and adaptive regulation of physiological functions mediated by the sympathetic branch [21]. The increase in renin is responsible for the action of the sympathetic-adrenal system and the stimulation of adrenergic receptors by adrenaline and noradrenaline, and as a consequence, the reduction in transmural pressure in the afferent arterioles [22]. A cornerstone in understanding the processes regulating sodium metabolism is the knowledge of the role of hormones (Table 1). The release of renin activates a cascade of events. Angiotensin II acts via the AT1 and AT2 receptors [4]. This hormone, acting on the subcutaneous membrane, activates Na^+^-K^+^ ATPase and moves Na^+^ ions into the interstitial space, reducing their excretion and increasing water reabsorption [23]. Aldosterone is a key hormone that helps maintain the proper concentration of both sodium and potassium in the body. Its receptors can be found in the kidneys, circulatory system, and brain, and its action is to increase the rate of transcription of genes responsible for the synthesis of Na^+^-K^+^ ATPase and ENaC channels and to intensify the effect of Na^+^-H^+^ countertransport [4]. Another important factor that determines sodium concentration in the body is the ADH. The increase and inhibition of ADH secretion occur in response to changes in plasma osmolality [24]. An increase in plasma osmolality of just 2 mOsm/kg H_2_O stimulates the synthesis of ADH in the hypothalamus and its release from the posterior pituitary gland into the bloodstream. ADH acts in the kidneys via V2 receptors (distal tubules and collecting ducts) and V1 receptors in the renal vessels. V2 receptors, which are sensitive to ADH, are located in the collecting duct of the nephrons [21]. This activates adenylate cyclase, increases intracellular cAMP, and stimulates protein kinase A (PKA), leading to phosphorylation and apical membrane insertion of aquaporin-2 (AQP2) water channels. Enhanced *AQP2* expression increases tubular water permeability, allowing passive water reabsorption along the medullary osmotic gradient created by sodium chloride and urea [22]. This results in the formation of concentrated urine and conservation of body water during states of dehydration or reduced effective circulating volume [23]. Consequently, increased water retention lowers serum sodium concentration through a dilutional effect. Angiotensin II also stimulates vasopressin-secreting neurons [25]. The mechanism of action of vasopressin involves increasing passive water transport, which is dependent on osmotic concentration. A 2% increase in plasma osmolality causes a threefold increase in plasma vasopressin levels [25]. However, it may indirectly influence the increase in osmolality by synergistically acting with aldosterone via ENaC channels, thereby increasing Na^+^-K^+^-Cl^−^ co-transport and enhancing urea diffusion, which in turn increases osmolar production in the renal medulla and reduces blood flow through the renal vasa recta [26]. Natriuretic peptides play a key role in regulating sodium levels. Among them, ANP (A-type natriuretic peptide or atrial natriuretic peptide) and BNP (B-type natriuretic peptide or brain natriuretic peptide) occupy a special place, acting through the NPR1 receptor. These peptides act by increasing blood flow in the kidney [27]. They constrict the efferent vessels and dilate the vessels supplying blood to the glomeruli, thereby increasing the GFR. ANP boosts natriuresis by inhibiting sodium channels and Na^+^-K^+^-ATPase activity, as well as inhibiting renin release from granular cells [28].

### 1.2. Assessment of Hydration Status as a Clinical Challenge

Dehydration, most often associated with diarrhea and gastroenteritis, is an important pathway leading to death in these common disorders [29]. In these cases, we have guidelines and scales (e.g., the WHO scale for dehydration) that help us through the diagnostic and therapeutic processes. Table 2 shows the dehydration scale, which gathers critical clinical findings from patients and can be helpful in assessing their hydration status, including that of patients with electrolyte disorders. In children, TBW (total body water) accounts for approximately 65–80% of body weight (80% in infants and around 65% in older children), making neonates and infants more susceptible to dehydration.

Children experience higher insensible water losses than adults due to their larger body surface area-to-mass ratio; they lose more water per kilogram of body mass [30]. This phenomenon is further compounded by augmented transepidermal water loss due to diminished skin thickness and enhanced permeability, alongside elevated respiratory rates that engender greater evaporative losses from the airways [31]. It is important to note that insensible losses are mostly made up of free water, with no sodium loss at the same time. Consequently, children are particularly susceptible to rapid increases in plasma sodium concentration during dehydration, fever, or inadequate fluid intake. This reflects their narrower physiological buffering capacity and less efficient compensatory capacity compared with adults [32].

We have three categories of dehydration depending on the state of natremia: isotonic, hypotonic, and hypertonic [33]. Sodium is the key osmolyte in the human body. Most of it (about 91%) remains in the extracellular space [24]. Changes in the plasma levels of this electrolyte can be perceived as life-threatening and cause dramatic damage to the nervous system. Children under 16 years of age, women, athletes, and people suffering from SIAD (syndrome of inappropriate antidiuresis, formerly syndrome of inappropriate antidiuretic hormone release) or hypoxia are more susceptible to hyponatremia and its effects [34]. To maintain water balance, patients should receive an adequate amount of fluids (mL per kilogram of body weight) according to the Holliday-Segar method (in infants, breastfeeding should be taken into account) [35]. Mild dehydration may manifest itself only as decreased urine output or remain asymptomatic. In other cases, we may observe dryness of the oral mucosa, decreased skin tone, or tachycardia. A capillary refill time of >2 s is considered a sign of moderate-to-severe dehydration [36]. In infants, clinical evaluation of the frontal fontanelle is necessary, as a sunken fontanelle also indicates severe dehydration [37]. Neurological symptoms, like altered mental status, lethargy, vomiting, nausea, and headache, when present, indicate serious fluid imbalance and the need for rapid response [38]. Critical fluid imbalance ultimately leads to hypovolemic shock with the need for immediate fluid therapy [33].

**Table 2:** Clinical features of the child or infant used to determine the severity of dehydration—authors’ own modification based on the literature [29,30,31,32,33,34,35].

Clinical Feature		Slightly Dehydrated	Moderate Dehydration	Severe Dehydration
Body weight loss	Infants	5%	10%	15%
	Children	3%	6%	>9%
Pulse		Normal	tachycardia	tachycardia
Capillary return		<2 s	2–4 s	>4 s
Frontal fontanelles		Normal	normal	sunken
Oral mucus		Moistened	dry	dried out, chapped
Thirst		Normal	drinks eagerly, thirsty	drink poorly or be unable to drink
Blood pressure		Normal	normal	lowered
Urine Specific Gravity		<1020	>1020	Anuria
Diuresis			Oliguria	anuria
General condition		Calm, conscious	Restlessness, agitated	Unconscious, sleepy, flaccid
Tears		Normal	absence	absence
Eyes		Normal	sunken	Sunken
Skin fold		Goes back quickly	Goes back slowly	Goes back very slowly

## 2. Hyponatremia

Since sodium imbalance is one of the most common electrolyte disorders in children [39] (e.g., 17.9% of patients in emergency departments [38]), it may be surprising that it is most often diagnosed incidentally [40]. Sodium level can be considered the most important and critical in laboratory tests, being at the same time one of the standard tests. Delays in treatment may lead to cerebral edema and are associated with a high risk of poorer prognosis and mortality. Hyponatremia is defined as a plasma sodium concentration of less than 135 mmol/L (normal range 135–145 mmol/L) [38]. The time of onset of hyponatremia allows for classification into two categories: acute and chronic. By acute, we mean when it starts within 48 h, while chronic is defined as occurring beyond this time. Based on sodium levels, we can classify hyponatremia as mild (130–135), moderate (125–130), and severe (<125) [38]. Routine laboratory testing involves measuring the concentration of sodium in plasma. Total sodium levels in other tissues and body fluids, e.g., whole blood, should not be used because they are usually underestimated by 2–4 mmol/L [40]. The sodium range indicates changes in plasma osmolality and is related to blood volume. Sodium, urea, and glucose, as main osmolytes, are used to calculate plasma osmolality using the formula [41]:

Osmolality = 1.86 (Na^+^ mmol/L) + glucose (mg/dL)/18 + serum (blood) urea nitrogen (mg/dL)/2.8

This equation leads to further clinical subdivisions into hypertonic (>300), isotonic (285–300), hypotonic (<285), and, even more importantly, volume-related euvolemic, hypervolemic, and hypovolemic [38]. Mild hyponatremia is usually asymptomatic [40]. Clinical symptoms typically appear when the serum sodium falls below 120 and vary with the duration and severity of the decline. Earlier symptoms may occur when the sodium level changes rapidly. As a result of a sharp drop in sodium levels, primarily neurological symptoms are observed (a drop below 120 can lead to seizures, brain herniation, dementia, and even death) [38]. We can also observe hyponatremic encephalopathy. The most common early symptoms are vomiting, nausea, headache, or impaired mental status. In more severe cases, we can observe neurological dysfunction with seizures, coma, impaired consciousness, respiratory arrest, or myocardial ischemia [40]. Importantly, differences in children’s anatomy make them more sensitive to changes in brain volume (a higher brain-to-skull ratio) because there is less space for brain tissue edema [38]. Due to these conditions, symptoms appear earlier and are more pronounced in individuals with higher sodium levels than in adults. Asymptomatic hyponatremia can occur when the amount of sodium in the plasma decreases gradually (due to adaptation of brain cells to maintain electrolyte balance), and the brain can counteract cerebral edema by secreting electrolytes and organic osmolytes. Increased intracranial pressure can lead to Ayus–Arieff syndrome, which manifests as noncardiogenic pulmonary edema with hypoxia and impaired regulation of brain volume secondary to cerebral edema [40]. Neurological changes in hyponatremia, especially chronic hyponatremia, may occur later due to adaptive mechanisms. To prevent brain damage in hyponatremia, cells release potassium and chloride ions from the intracellular space, thereby initiating osmotic changes and shifting the water content from the inside of the cells to the extracellular space. Also, after some time, brain cells, in response to changes in osmotic pressure in the extracellular fluid, start to extrude osmotically active organic substances from inside the cell (the best known are betaine, taurine, glutamate, and myo-inositol), counteracting unfavorable changes [42]. These transitions, although they are a defense mechanism, if they last too long, without proper sodium correction, may lead to seizures due to the outflow of osmotically active molecules such as GABA and glutamate to the extracellular space [43]. These slowly occurring adaptation processes, involving the removal of organic osmolytes and downregulation in the event of too rapid sodium repletion, predispose to ODS (osmotic demyelination process), primarily in the pons, but can also occur in other brain structures (EPM—extrapontine myelinosis), such as the basal ganglia [44]. Cells are unable to restore the osmolyte concentration lost during adaptation to the hypoosmotic environment at an appropriate rate, leading to damage to the blood–brain barrier. As a consequence, pro-inflammatory cytokines are released, microglia and the complement system are activated, and the induced inflammation leads to damage to the myelin sheaths [45]. In the treatment of hyponatremia, our goal should be to correct sodium concentration at an appropriate time to avoid serious complications [46] and prevent future decreases. The choice of therapeutic method depends on the duration of hyponatremia (acute or chronic) and the clinical symptoms presented by the patient [47]. In cases of chronic hyponatremia, the patient should have fluids gradually replaced using 0.9% NaCl, with particular emphasis on preventing complications of too rapid correction or 3% NaCl when severe symptoms occur. In these patients, due to the duration of symptoms, adaptation has most likely already occurred, and most often we do not observe symptoms, or the symptoms are mild [46]. The rate of fluid administration (presented in Table 3) depends on the severity of clinical symptoms.

**Figure 2:**
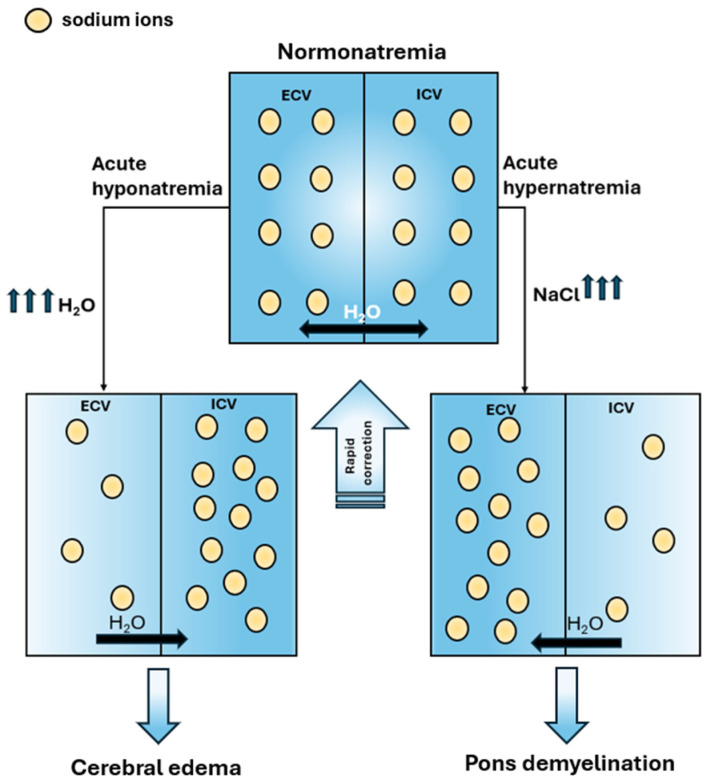
Central nervous system homeostasis under normonatremic conditions and pathophysiological changes occurring during acute hyponatremia and acute hypernatremia, along with their consequences. In this context, rapid correction of sodium imbalance restores normonatremia. ECV-extracellular volume; ICV-intracellular volume (brain cells).

In acute presentations, rapid intervention is essential, as abrupt elevations or reductions in serum sodium concentration may result in serious neurological complications, including pontine demyelination or cerebral edema, respectively (Figure 2) [46]. In cases of symptomatic acute hyponatremia with seizures, the fear of severe complications has a higher priority than the possible side effects of treatment. The type of fluid used in therapy and the rate of correction of sodium concentration in acute hyponatremia depend on the presence of clinical symptoms (Table 4).

**Table 4:** Proper sodium correction per day using the specified sodium solution, divided by the type of acute hyponatremia.

Symptom Severity	Type of Fluid Used in Therapy	Sodium Correction
Symptomatic	3% NaCL	3 to 5 mL/kg in 10–15 min in the initial phase, with a rise in serum sodium concentration of about 2.5 to 4 mmol/L
Asymptomatic	0/9% NaCl (no need to use hypertonic solution)	6 to 8 mmol/L over 24 h [45]

The proper amount of sodium we should calculate according to the formula:

SODIUM DEFICIENCY = (135 mmol/L − sodium level in the patient) × 0.6 (or 0.7 in infants) × body weight + basic sodium requirement 1–2 mmol/kg/24 h

**Figure 3:**
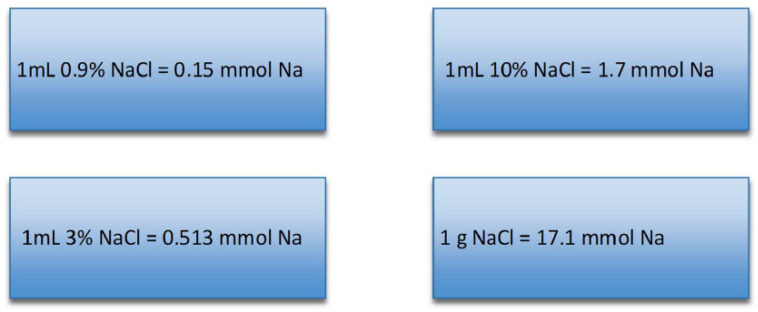
Content of mmol of sodium ions in one milliliter of solutions used in hospitals and in one gram of solid sodium chloride.

In clinical practice the proper amount of medication depends on the type of sodium concentration in the medication used (presented in Figure 3), and the control laboratory test serum sodium level is checked within every 2–3 h.

**Figure 4:**
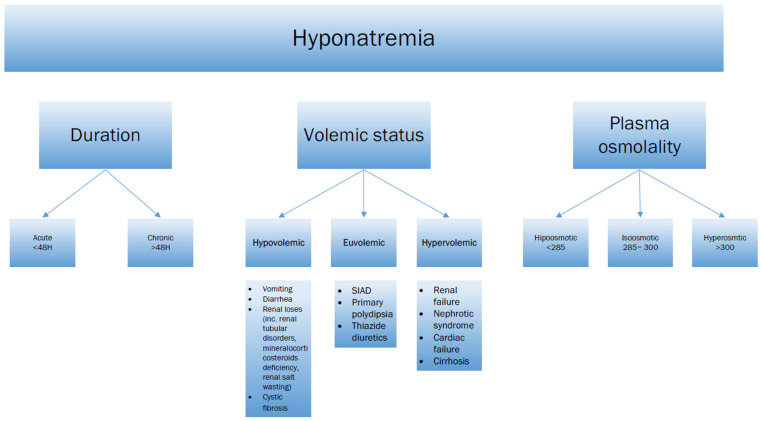
Possible classification of the hyponatremia depending on duration, volemic status, plasma osmolality, and their etiology.

Possible classification of the hyponatremia depending on duration, volemic status, plasma osmolality, and their etiology were depicted in Figure 4.

### 2.1. SIAD

By definition, it is an uncontrolled, excessive release of AVP from the pituitary gland or other sources, such as tumors, that is inadequate to the plasma osmolality and blood volume. In healthy patients, AVP secretion is usually controlled by increased osmolality, changes in blood volume, and blood pressure [48]. Clinical symptoms occur due to excessive action on V2R receptors in the renal tubules by AVP [49]. An increase in plasma osmolality above 280 mOsm/kg causes the release of AVP. In children, a larger amount of the hormone is released at the same plasma osmolality as in adults. In patients with SIAD, AVP excretion is higher than expected based on changes in plasma osmolality in healthy patients [50]. Hyponatremia with isovolemia is characteristic of SIAD. In the literature, four types of SIAD are distinguished—A, B, C, and D. The first, type A, is caused by dysregulation of AVP secretion. Type B means normal AVP with preserved normal osmolality. Type C—“osmostat reset”—means damage to the osmotic center of the hypothalamus. The last, type D, means that we have a genetic cause of the lack of hormone. In type D, the changes are associated with activating a pathogenic variant of the *AVPR2* gene located, as already mentioned, on chromosome X, so it naturally occurs more often in men [51]. Some widely used medications can cause secondary SIAD, e.g., selective serotonin reuptake inhibitors, anticonvulsants, antipsychotics, other antidepressants, or even painkillers [50]. The clinical symptoms of SIAD result from hyponatremia and its severity, so they do not differ from those described in the previous paragraph. The therapeutic process usually begins with fluid restriction or is combined with pharmacotherapy (antagonists of V2 receptors—vaptans). However, it should be remembered that in the case of hyponatremia, it may be necessary to include salt solutions (e.g., 3% NaCl solution) due to possible neurological complications [50].

### 2.2. Cerebral Salt Wasting Syndrome

One of the causes of hyponatremia is cerebral salt-wasting syndrome (CSWS). The cause of this disease is not yet fully understood. So far, we know a few facts. First, it has been observed in children with brain injuries. Several studies have shown a coincidence of brain damage and hyponatremia. The most significant risk of hyponatremia syndrome concerns the group of children after brain surgery, SAH (subarachnoid hemorrhage), and meningitis. It has also been observed after ketamine infusion, medulloblastoma, epilepsy, Kawasaki disease, lissencephaly, or hematopoietic stem cell infusion. It appears to occur more frequently in younger children (0–3 years), particularly in males [51]. In some cases, it may be related to vasodilator drugs, but the mechanism remains unclear. One hypothesis is that damage to the central nervous system leads to reduced sympathetic stimulation of the proximal renal tubules, resulting in excess natriuresis, hypovolemia, and hyponatremia due to changes in the excess production of natriuretic peptides. This can be explained as a protective mechanism in subarachnoid hemorrhage; this process results in a reduced blood volume and decreased bleeding [51]. The second theory is that mechanical disruption of the blood–brain barrier helps brain natriuretic peptide (BNP) enter the bloodstream. This hormone acts on the collecting tubules in two ways. First, sodium reabsorption is inhibited. The second reason is that BNP affects the tubules by reducing renin release. In CSWS, an increased rate of uric acid and urea excretion may be observed, coexisting with impaired sodium reabsorption in the proximal tubules. The diagnosis of CSWS requires preserved renal function and the hypothalamic–pituitary–adrenal axis [48]. In this type of hyponatremia, we usually find hypovolemia, but when secondary AVP resistance increases, it can lead to euvolemia. In the diagnostic process, we can see typical symptoms of hypovolemic dehydration (dry mucus, tachycardia, weight loss, negative fluid balance, decreased skin turgor). There are some laboratory test results that are typical for CSWS. We may observe increased BUN, serum creatinine, and hematocrit and decreased serum uric acid, serum osmolality, and urine sodium excretion [51]. Additionally, the sodium concentration in urine typically exceeds 40 mmol/L, and the urine osmolality is higher than 100 mOsmol/kg. This diagnostic laboratory panel, along with clinical signs of dehydration, is crucial for evaluating CSWS. During the diagnostic process, we should exclude other potential causes of hyponatremia, such as kidney disease and polydipsia. We must emphasize the importance of differentiating between CSWS and SIAD, as they require different treatment procedures. SIAD may appear similar to CSWS in laboratory test results. This disease entity is differentiated by the volume of blood in the venous bed, which is normal or higher in SIAD [51]. The basis of treatment for CSWS is treating the underlying cause, which is most often central nervous system trauma [52]. In clinical practice, we should also focus on correcting hyponatremia with 0.9% NaCl or a 3% NaCl solution, depending on the clinical outcomes [53]. Saline treatment may be ineffective in some patients. If this approach proves ineffective, fludrocortisone, a mineralocorticoid with proven antinatriuretic properties, may be added to the therapy [54].

## 3. Hypernatremia

Hypernatremia is a sodium level above 145 mmol/L. It is less common than hyponatremia, but it is also essential for early diagnosis because of potential serious brain damage [55]. In the initial phase, water moves from the intracellular to the extracellular space. Dehydrated brain cells begin to accumulate sodium, potassium, chloride, and osmolytes. This mechanism changes the water content of cells in chronic hypernatremia and maintains turgor in cells [56]. Healthy kidneys begin to produce concentrated urine when sodium levels rise. The volemic status indicates the cause of this dyselectrolytemia. Hypovolemia is primarily caused by extrarenal fluid loss or inadequate water intake. Hypervolemia occurs when sodium intake is immoderate. Moderate renal or extrarenal fluid loss manifests as isovolemic. Hypernatremia is associated chiefly with dehydration secondary to gastrointestinal disorders or infection [55]. Also, water deficiency, excessive salt intake, and overproduction of mineralocorticoids can lead to hypernatremia. It may also be the result of urine concentration defects [55]. In this condition, we can observe elevated sodium levels within minutes and peaks within hours. Hypernatremia is characterized by high mortality, especially when the plasma sodium level exceeds 190 mmol/L (50% mortality) [57]. Children and young adults are at higher risk of hypernatremia (low mass to surface area ratio) [58]. The most common symptoms are nonspecific, originating from the nervous system, such as nausea, vomiting, irritability, restlessness, weakness, or fever. In severe cases, symptoms such as spasticity, convulsions, seizures, or coma may occur [57]. Rapidly increasing sodium levels can cause intracranial hemorrhage, venous sinus thrombosis, or demyelination [40]. Hypernatremia is also associated with muscle problems such as rhabdomyolysis [57]. Renal failure combined with an inability to communicate the need for fluids (as in infants) can easily lead to hypernatremia. Correction of plasma sodium concentration, as in the case of hyponatremia, should be performed gradually due to the possibility of cerebral edema, seizures, or other neurological complications. The literature most often recommends correction by <0.5 mmol/L per hour or by <10–12 mmol/L per 24 h [59]. To evaluate water deficit in patients with hypernatremia caused by dehydration, the following formula can be used:

4 mL × weight (kg) × desired change in serum sodium (mmol/L)

It is essential to modify the procedure in patients with urine concentration disorders, such as AVP resistance, where the use of hypotonic fluids is recommended [60].

**Figure 5:**
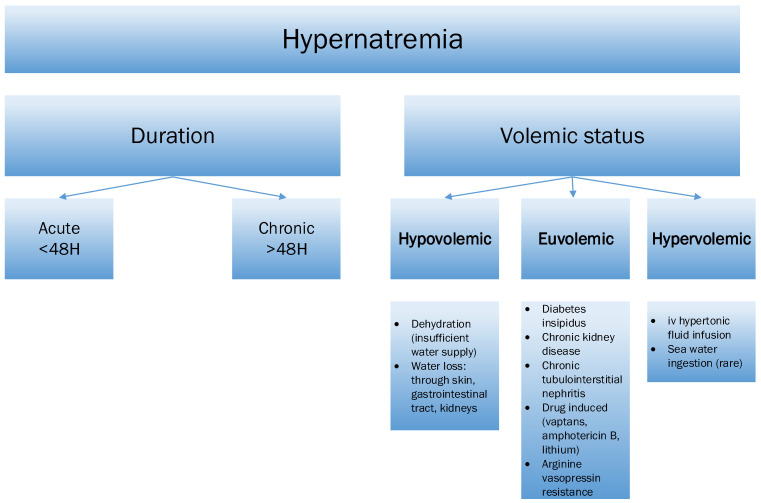
Possible classification of the hyponatremia depending on the duration and volemic status and their etiologies.

Possible classification of the hyponatremia depending on the duration and volemic status and their etiologies were depicted in Figure 5.

### 3.1. Central Diabetes Insipidus

It is a complex disease of various origins, most often caused by permanent or temporary inefficiency of secretion or disturbances in the transport of antidiuretic hormone (thus, at present, the proposed name of the entity is AVP deficiency—AVD) [61]. The hormone responsible for AVD is ADH (antidiuretic hormone), produced in the hypothalamus, stored in the posterior pituitary gland, and affecting the receptors of the renal tubules. ADH secretion is controlled by osmoregulation and baroreceptors. This mechanism, involving two negative feedback loops, maintains the water-sodium balance in the human body. Even small changes in plasma osmolality, less than 1%, stimulate hypothalamic receptors that initiate osmoregulatory mechanisms. Once released from the hypothalamus into the bloodstream, ADH binds to AQP2 channels in the renal tubules and begins to concentrate urine. Inefficient production of ADH by pituitary cells is one of the causes of AVD [62]. AVD is most often caused by congenital (genetic) and secondary hypothalamic disorders, e.g., hypoxia, trauma after neurosurgical surgery, and tumors [63]. The inability to concentrate urine is manifested by the excess production of insufficiently concentrated urine. Rapid urine excretion is the result of a decrease below 10–15% of normal AVP secretion. Earlier clinical symptoms may be invisible due to effective compensatory mechanisms [63]. Symptoms of AVD, such as polydipsia, polyuria, or nocturia, are similar to those of diabetes mellitus and should be considered in the differential diagnosis. Symptoms can be divided into two groups. The first are those resulting from dehydration—hypotension, rarely acute tubular necrosis, renal ischemia, and hypovolemic shock. Additionally, the patient may suffer from gastrointestinal symptoms (vomiting, constipation), fever, irritability, and poor quality of sleep. We may also find growth retardation. The second is caused by hyperosmolality. These are primarily neurological problems resulting from cerebral dehydration and osmotic changes in neurons. Symptoms can be highly diverse and vary in severity. This can range from mild disorientation to, in the worst cases, seizures, coma, cerebral infarction, or focal neurological deficits. Patients with AVD may be at risk of SAH or deep vein thrombosis. We should also be aware of the possibility of dilatation of the collecting systems and bladder due to massive urine output [63]. Making a diagnosis requires confirmation of clinical results in laboratory tests. AVD is characterized by a sodium level above 145 mmol/L, an osmolality above 300 mOsm/kg H_2_O in blood, and a simultaneously decreased urine osmolality below 600 mOsm/kg H_2_O. To confirm the diagnosis, a water deprivation test is needed [64]. The treatment of choice is replacement of vasopressin analogs. Synthetic analogues are 2000–3000 times weaker than endogenous vasopressin. The half-life of analogues is up to 3.5 h. The effect should be visible after 1–2 h and last from 6 to 18 h [65].

### 3.2. Arginine Vasopressin Resistance

Arginine vasopressin (AVP) resistance (AVR) (formerly known as nephrogenic diabetes insipidus) by definition is a congenital or acquired defect in the distal and collecting tubules that results in insufficient urine concentration due to insensitivity to AVP. Most often, these are acquired forms, but congenital ones may be more dangerous. In infants, polyuria and the tendency to dehydration are extreme, and when combined with the inability to signal the need for hydration, may pose a critical risk [66]. *AVPR2* pathogenic variants occur in nearly 90% of patients with hereditary (X-linked recessive) disease. The remaining 10% of genetic mutations in DNA are almost entirely due to pathogenic variants in the *AQP2* gene, which is inherited in an autosomal-dominant manner. Acquired cases may be the result of drugs that reduce *AQP2* expression [66]. One of the most common drugs causing AVR is lithium salts, widely used in psychiatry, but it may also be foscarnet, cidofovir, amphotericin B, or cisplatin [67]. Acquired AVR can be a consequence of other systemic diseases affecting the renal medulla, such as sarcoidosis, Sjogren’s syndrome, cystic kidney disease, amyloidosis, or multiple myeloma [67]. Severe electrolyte disturbances, such as hypokalemia and hypercalcemia, require careful observation as potential causes of AVR [64]. The difference between congenital and acquired forms is the presence of growth disorders characteristic of the congenital form of the disease. Polyuria and polydipsia are more common in acquired forms of the disease, which usually manifest themselves later [67]. In acquired forms of AVR, treatment is based on treating the underlying disease or modifying the treatment. The next step is to alter the diet to a low-protein one and limit sodium intake. Pharmacological treatment of congenital AVR includes hydrochlorothiazide (reduces urine output by about 50%) [64].

Although researchers have made substantial progress in elucidating the genetic basis of arginine vasopressin resistance, the continued identification of novel pathogenic *AVPR2* variants presenting from the neonatal period onward indicates that researchers have not yet fully defined the molecular architecture of this disorder in children [68]. This emphasizes the importance of expanding and refining pediatric-specific genetic variant databases in future research.

Of interest, several experimental and translational studies suggest that, alongside classical vasopressin-mediated trafficking and expression of *AQP2*, post-transcriptional regulators such as microRNAs can influence *AQP2* levels and activity [23,69].

This multi-layered regulatory architecture opens a promising avenue for future research, particularly in children, where developmental differences may influence these pathways and novel microRNA signatures could serve both mechanistic and biomarker roles by characterizing specific microRNAs involved in *AQP2* regulation.

Table 5 provides a concise summary of the clinical manifestations and outcomes of the diseases discussed above, which may present with disturbances of sodium and water homeostasis and should therefore be considered in the differential diagnosis.

## 4. Conclusions

Sodium, although present in the body in small amounts, plays a key role in major life processes and is the principal cation of extracellular fluids. The brain and kidneys, and the active substances they secrete, play the most important role in regulating sodium metabolism—together, this interplay can be referred to as the sodium brain-kidney axis. In daily medical practice, disorders of sodium metabolism are the most common ionic disorders, although they are often detected incidentally. Severe sodium disorders can lead to irreversible changes in the central nervous system and even death.

In any patient with a sodium disorder, the state of goiter should be determined, as water and sodium are inextricably linked. Whenever possible, the underlying cause of the disorder should be identified and treated appropriately. In acute presentations, rapid intervention is essential, as abrupt elevations or reductions in serum sodium concentration may result in serious neurological complications, including pontine demyelination or cerebral edema, respectively. In contrast, in chronic disorders, a cautious and gradual correction is recommended; overly rapid normalization may provoke neurological injury similar to that observed with inadequate correction in acute settings.

The differential diagnosis of hypernatremia should include dehydration, gastrointestinal or renal water losses, arginine vasopressin deficiency and resistance, and excessive sodium chloride intake. In cases of hyponatremia, potential etiologies include renal or gastrointestinal sodium loss (e.g., vomiting), cutaneous losses, and endocrine or systemic disorders such as glucocorticoid or thyroid hormone deficiency, cerebral salt-wasting syndrome, syndrome of inappropriate antidiuresis, heart failure, or nephrotic syndrome.

A thorough understanding of the underlying pathophysiological mechanisms, combined with a structured diagnostic pathway and knowledge of therapeutic principles—shared across conditions yet requiring individualized application—facilitates accurate diagnosis and optimal management in complex clinical scenarios.

## Figures and Tables

**Table 1:** The main hormones regulating sodium homeostasis in the human body.

Hormone	The Place of Hormone Production	Receptors	Hormone-Releasing Factors	Hormone Action	Influence on Sodium Concentration in Plasma
**Renin**	Kidneys (juxtaglomerular apparatus)	Prorenin receptors	Drop in sodium concentration in plasma A decrease in urinary sodium Drop in blood pressure Decreased renal perfusion pressure Reduction in extracellular fluid volume	RAA system activation	Na ↑
**Angiotensin**	Prohormone converted in the lungs by renin	AT1 (angiotensin), AT2	Drop in blood pressure Drop in sodium concentration in plasma A decrease in urinary sodium Decreased blood volume Increased renin concentration, increased sympathetic activation	Vasoconstriction (via its receptors in the arteriole bed)	Na ↑
**Aldosterone**	Cells of the adrenal glomerular layer	MR (mineralocorticoid) receptors	RAA system activation High potassium concentration ACTH increased level Stress Drugs (e.g., ACE inhibitors)	Increases the absorption of sodium and water in the renal tubules	Na ↑
**ANP and BNP**	Muscle cells of the atria of the heart (ANP), muscle cells of the ventricles of the heart (BNP)	NPR1 (natriuretic receptor peptide)	Stretching of the heart’s atria/ventricles by blood High levels of angiotensin and aldosterone	Constriction of the efferent vessels and widening of the vessels supplying blood to the glomeruli Increasing blood flow in the kidneys	Na ↓
**Vasopressin**	Hypothalamus	V1 (in arteries), V2 (in kidneys), V3	Low blood pressure Low plasma osmolality Low blood volume Naturally secreted during sleep	Increasing passive water transport in renal tubules	Na ↓ (indirectly by water retention in the body)

Black arrows next to “Na” indicate an increase or decrease in plasma sodium concentration.

**Table 3:** Proper sodium correction per day using specified sodium solution divided by type of chronic hyponatremia.

Type of Chronic Hyponatremia	Type of Fluid Used in Therapy	Sodium Correction per Day
Asymptomatic	0.9% NaCl	6 to 8 mmol/L
Mild to moderate	0.9% NaCl	6 to 8 mmol/L
Severe (<120 mmol/L)	3% NaCl	6 to 8 mmol/L (3–5 mL/kg in the initial phase to increase serum sodium rapidly by 2.5 to 4 mmol/L)

**Table 5:** Clinical manifestation and output of the diseases presented in this review in the context of differential diagnosis with psychogenic polydipsia.

	Psychogenic Polydipsia	Arginine Vasopressin Deficiency	Arginine Vasopressin Resistance	Syndrome of Inappropriate Antidiuresis	Cerebro-Renal Salt Wasting Syndrome
**Mechanisms**	Excess water intake	Lack of endogenous vasopressin (good response to exogenous hormone)	Vasopressin insensitivity	Change in osmotic set point to start the release of ADH	Excess amount of natriuretic peptides, excess loss of sodium and water
**Sodium serum level**	↓	↑/=	↑/=	↓	↓
**Serum osmolality**	↓	↑ (>300)	↑ (>300)	↓ (<280)	↓ (<280)
**Blood pressure**	=	=/↓	=/↓	=/↑	=/↓
**Heart rate**	=	=/↑	=/↑	=	=/↑
**Urine output**	↑	↑	↑	=/↓	↑
**Urine sodium concentration**	↓	↓	↓	↑	↑↑↑
**Urine osmolality**	↓	↓	↓	↑	↑
**Urine specific gravity**	↓	↓	↓	↑	↑
**Water deprivation test**	Urine osmolality >750 (800)	Urine osmolality <750 (800)	Urine osmolality <750 (800)	No diagnostic value	No diagnostic value
**Vasopressin test**	No diagnostic value	Urine osmolality >750 (800)	Urine osmolality <750 (800)	No diagnostic value	No diagnostic value

↑ increase; ↓ decrease; = no change; ↑↑↑, pronounced increase.

## Data Availability

No new data were created or analyzed in this study. Data sharing is not applicable to this article.
